# Innovative Discrete Multi-Wavelength Near-Infrared Spectroscopic (DMW-NIRS) Imaging for Rapid Breast Lesion Differentiation: Feasibility Study

**DOI:** 10.3390/diagnostics15091067

**Published:** 2025-04-23

**Authors:** Jiyoung Yoon, Kyunghwa Han, Min Jung Kim, Heesun Hong, Eunice S. Han, Sung-Ho Han

**Affiliations:** 1 Department of Radiology and Research, Institute of Radiological Science, Yonsei University College of Medicine, Seoul 06134, Republic of Korea; 2 CTO Division, Olive Healthcare, Seoul 07796, Republic of Korea

**Keywords:** DMW-NIRS, non-invasive diagnostic tools, breast lesion differentiation, BI-RADS category 4A lesions, adaptive BI-RADS modeling

## Abstract

**Background/Objectives**: This study evaluated the role of a discrete multi-wavelength near-infrared spectroscopic (DMW-NIRS) imaging device for rapid breast lesion differentiation. **Methods**: A total of 62 women (mean age, 49.9 years) with ultrasound (US)-guided biopsy-confirmed breast lesions (37 malignant, 25 benign) were included. A handheld probe equipped with five pairs of light-emitting diodes (LEDs) and photodiodes (PDs) measured lesion-to-normal tissue (L/N) ratios of four chromophores, THC (Total Hemoglobin Concentration), StO_2_, and the Tissue Optical Index (TOI: log10(THC × Water/Lipid)). Lesions were localized using US. Diagnostic performance was assessed for each L/N ratio, with subgroup analysis for BI-RADS 4A lesions. Two adaptive BI-RADS models were developed: Model 1 used TOI_L/N_ thresholds (Youden index), while Model 2 incorporated radiologists’ reassessments of US findings integrated with DMW-NIRS results. These models were compared to the initial BI-RADS assessments, conducted by breast-dedicated radiologists. **Results**: All L/N ratios significantly differentiated malignant from benign lesions (*p* < 0.05), with TOI_L/N_ achieving the highest AUC-ROC (0.901; 95% CI: 0.825–0.976). In BI-RADS 4A lesions, all L/N ratios except Lipid significantly differentiated malignancy (*p* < 0.05), with TOI_L/N_ achieving the highest AUC-ROC (0.902; 95% CI: 0.788–1.000). Model 1 and Model 2 showed superior diagnostic performance (AUC-ROCs: 0.962 and 0.922, respectively), significantly outperforming initial BI-RADS assessments (prospective AUC-ROC: 0.862; retrospective AUC-ROC: 0.866; *p* < 0.05). **Conclusions**: Integrating DMW-NIRS findings with US evaluations enhances diagnostic accuracy, particularly for BI-RADS 4A lesions. This novel device offers a rapid, non-invasive, and efficient method to reduce unnecessary biopsies and improve breast cancer diagnostics. Further validation in larger cohorts is warranted.

## 1. Introduction

Breast cancer is a rapidly increasing global health concern, underscoring the need for precise differentiation between malignant and benign breast lesions [1]. The Breast Imaging Reporting and Data System (BI-RADS) provides standardized guidelines for categorizing breast lesions. According to these guidelines, BI-RADS Category 3 lesions, which have a malignancy likelihood of 2% or less, do not require biopsy but are recommended for a 6-month follow-up examination. In contrast, BI-RADS Category 4A lesions, with a malignancy likelihood of 2–10%, necessitate biopsy [2]. Unfortunately, this often leads to an increased number of unnecessary biopsies with false positive results, resulting in inconvenience and escalated examination costs for patients with benign lesions. Therefore, there is a need for additional ancillary diagnostic tools to more accurately characterize breast lesions and address the limitations of conventional imaging methods.

One promising functional imaging technology is diffuse optical spectroscopic imaging (DOSI), which employs near-infrared (NIR) light to evaluate tissue hemodynamics and biochemical composition. DOSI enables quantification of tissue oxy- and deoxy-hemoglobin, water content, and lipid concentration, providing valuable insights into tissue perfusion and metabolism [3,4,5]. Clinical research has explored various DOSI methods for breast cancer detection, diagnosis, and management. Recent applications include monitoring chemotherapy response [6,7,8,9,10], assessing breast density [11,12], identifying tumors in dense breast tissue, and aiding in the differential diagnosis of breast lesions [13,14,15,16,17], with ongoing developments in handheld NIR imaging probes further enhancing portability and clinical applicability [18].

In this study, we implemented a newly developed, commercially available DMW-NIRS device into clinical practice. This device uses discrete multi-wavelength LED and PD pairs to perform rapid and multi-channel measurements, presenting results on site. Its design and efficiency make it suitable for potential clinical applications in breast cancer diagnostics. The purpose of this study was to evaluate the role of this novel device in improving the accuracy of distinguishing malignant from benign breast lesions.

## 2. Materials and Methods

The DMW-NIRS device in rapid and multi-channel measurement (SenoVue, Olive Healthcare Inc., Seoul, Republic of Korea) was approved by the Ministry of Food and Drug Safety in Korea, and the protocol of this prospective study using the above device was issued by the Institutional Review Boards of Severance Hospital, Yonesi University (IRB approval No: 1-2019-0050, approval date: 4 August 2021). Informed consent was obtained from all participants prior to their inclusion in this study.

### 2.1. Study Population

**Figure 1:**
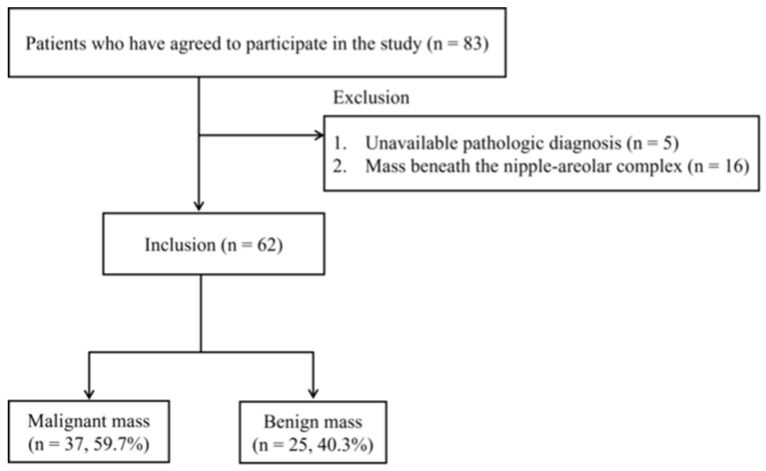
Flowchart of patient selection.

Women aged 20 years or older with breast lesions categorized as BI-RADS 3, 4, or 5 based on breast US were eligible for inclusion in this study. Exclusion criteria included pregnant or breastfeeding women, those with a history of significant breast trauma or inflammation, individuals who had undergone breast removal surgery, women with breast implants, or those with a history of photosensitivity. A total of 83 patients meeting the inclusion criteria agreed to participate and provided informed consent. Patients without an available pathological diagnosis (*n* = 5) or with breast masses beneath the nipple-areolar complex (NAC) (*n* = 16) were excluded. Ultimately, 62 women (age range: 29–80 years; mean age: 49.9 years) with 62 breast lesions (37 malignant and 25 benign) confirmed via US-guided biopsy were included in the analysis (Figure 1).

### 2.2. Evaluation of Clinical and Lesion Characteristics

All participants aged 40 years or older underwent mammography and breast US examinations as part of the diagnostic evaluation, while patients younger than 40 years underwent only breast US. Clinical information, including age, BMI, family history of breast cancer, history of hormonal therapy, and menopausal status, was collected via surveys.

Breast US examinations were performed by five breast-dedicated radiologists with 2–20 years of subspecialty training in breast imaging. They recorded the following parameters: mammographic density of the breast tissue based on BI-RADS criteria (only for patients who underwent both mammography and breast US), maximal tumor diameter, distance from the nipple, distance from the skin (measured from the top skin margin to the tumor’s most superficial site), breast thickness at the tumor site (measured from the top skin margin to the superficial margin of the pectoralis major muscle), and final BI-RADS category.

Pathologic diagnosis was determined through US-guided percutaneous core needle biopsy. Lesions categorized as BI-RADS Category 4 or 5 were biopsied according to BI-RADS recommendations. In cases categorized as BI-RADS Category 3, only the lesions that were biopsied based on patients‘ request were included. For lesions with prior pathological results within 2 years, additional biopsies were not performed.

### 2.3. Instrumentation and Measurement Procedure

DMW-NIRS measurements were conducted using the developed medical device, SenoVue (Olive Healthcare Inc.). SenoVue approaches the chromophore concentration of soft tissue but does not use expensive and complex equipment like existing frequency-domain or time-domain DOS instruments, and just uses continuous-wave measurement data from multi-wavelength LED and PD. The novelty of the DMW-NIRS of SenoVue is that it can directly obtain the concentrations of THb, water, and lipid from reflectance values by using machine learning methods without requiring any complex calculation processes that other devices do to separate scattering coefficients. Since it only requires a simple model prediction process using reflectance values of several wavelengths essential for THb, water and lipid, it only takes a few seconds from the end of the measurement to output the concentration results of the measurement area.

SenoVue consists of a main body and a probe connected with the main body via WI-FI. This handheld probe consists of five channels spaced 1 cm apart in a row, and LED-PD separation of each channel is 3 cm.

Lesion locations were marked by breast-dedicated radiologists based on conventional US findings. The DMW-NIRS probe was then placed at the marked lesion site with the patient in a supine position, while corresponding contralateral (normal) breast areas were measured for comparison.

Five channels operate sequentially in one measurement, taking reflectance values for eight wavelengths (688 nm, 795 nm, 808 nm, 830 nm, 860 nm, 915 nm, 975 nm, and 1064 nm) at each channel. Each wavelength also operates sequentially and the total operating time for 8 wavelengths is about 1.2 s. Optical power ranges between 0.5 and 3 mW depending on the wavelength.

**Figure 2:**
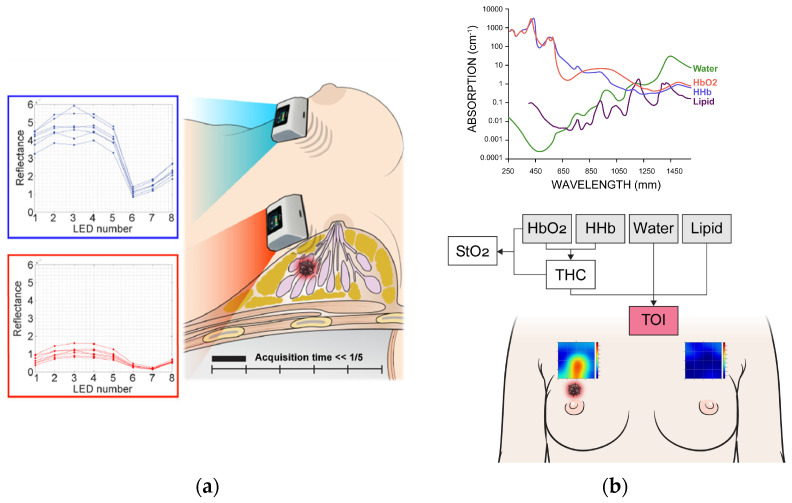
Measurement process (**a**) and spectral analysis (**b**) using discrete multi-wavelength near-infrared spectrum (DMW-NIRS). HbO_2_ = oxy-hemoglobin, HHb = deoxy-hemoglobin, Lipid = bulk lipid, StO_2_ = percent oxygen saturation, THC = total hemoglobin concentration, TOI = tissue optical index.

The measurement grid covers a 5 × 5 cm^2^ area. The probe was shifted by 1 cm in the downward direction within this region so the total 25 grid points covered this area for both sides of breasts. The entire scan process required approximately less than five minutes. If the NAC fell within the measurement area, the patient was excluded from the study. For lesions that were clearly visible, the region of interest (ROI) was selected based on the 5 × 5 image. For cases where lesion ROI selection was challenging, the central 9 spots were chosen, as measurements were centered on the lesion’s reference point. Figure 2 illustrates the measurement process and an example of the obtained data.

### 2.4. Data Analysis

The measured reflectance values for each wavelength were calibrated using phantom calibration process. Then, a series of calculations obtained the concentrations of oxy-hemoglobin (HbO_2_), deoxy-hemoglobin (HHb), water (Water), bulk lipid (Lipid), total hemoglobin concentration (THC = HbO_2_ + HHb), percent oxygen saturation (StO_2_ = HbO_2_/THC), and the tissue optical index (TOI = log10(THC × Water/Lipid)) [5] from those 8 wavelength reflectance values (Figure 2).

Lesion-to-normal ratios (L/Ns) of these quantitative values (HbO_2_, HHb, THC, StO_2_, Water, Lipid, and TOI) were calculated by dividing the mean value measured in the lesion ROI by the mean value measured in the corresponding contralateral normal breast ROI. The normal ROI was defined as the mirror location of the lesion ROI in the contralateral (unaffected) breast. For both the lesion and the normal region, multiple measurement spots were used, and the mean value of those spots was used for the ratio calculation. The L/N ratio was calculated using the following formula:

$$\frac{\sum_{i=1}^{n}The\;value\;of\;the\;i-th\;lesion\;spot\;in\;the\;ROI/n}{\sum_{j=1}^{n}The\;value\;of\;the\;j-th\;lesion\;spot\;in\;the\;normal\;ROI/n}$$

In particular, the concentrations of Water, Lipid, THC, and TOI at each measurement location were estimated using machine learning-based prediction models. These models were developed prior to this study and used the reflectance values of eight wavelengths—measured from various soft tissue regions using SenoVue—as predictor variables. As ground truth, the corresponding concentrations obtained from a DOSI device at the same tissue locations were used as response variables. Each chromophore-specific model was trained using a deep feedforward neural network with approximately 20,000 to 30,000 data points. The model performance showed high accuracy, with R^2^ values exceeding 0.99 for the training set and 0.94 for the test set (Figure S1).

### 2.5. Statistical Analysis

Clinical and lesion characteristics were compared between the malignant and benign groups using the chi-squared or Fisher’s exact tests. The L/N values of chromophores were compared using the Wilcoxon rank sum test, and box plots were used to visualize the distribution of L/N values between the two groups. Diagnostic performance metrics, including sensitivity, specificity, accuracy, and area under the receiver operating characteristic curve (AUC-ROC), were calculated for each L/N ratio. Subgroup analysis was specifically performed for BI-RADS Category 4A lesions.

Additionally, two adaptive BI-RADS categories were determined:

1. Model 1: BI-RADS categories were adjusted using TOI_L/N_ cutoff values determined by the Youden index. Lesions exceeding the threshold retained their original BI-RADS category, while those below the threshold were downgraded.
2. Model 2: This model integrates radiologists’ retrospective reassessments of US images with the DMW-NIRS results. The radiologists who initially performed the prospective BI-RADS assessments did not reference the DMW-NIRS results during their initial evaluations. Therefore, to develop Model 2, two breast-dedicated radiologists (M.J.K and J.Y) independently reviewed the previously captured US images and reassessed the BI-RADS categories based on their judgment. After this, they incorporated the DMW-NIRS results to refine their assessments and establish the second adaptive BI-RADS categories.

The AUC-ROCs of the adaptive BI-RADS models were calculated and compared to the original BI-RADS categories determined without DMW-NIRS assistance.

All statistical analyses were performed using R program (4.3.3, Foundation for Statistical Computing, Vienna, Austria). *p* values less than 0.05 were considered statistically significant.

## 3. Results

### 3.1. Clinical and Lesion Characteristics

Table 1 summarizes the comparison of clinical and lesion characteristics between malignant and benign groups. Among clinical characteristics, age and menopausal status were significantly different between two groups (both *p* < 0.05). Regarding lesion characteristics, maximal tumor diameter, distance from the nipple, distance from the skin, and BI-RADS category were significantly different between two groups (all *p* < 0.05).

In the subgroup analysis of BI-RADS Category 4A cases, only age showed a significant difference between the malignant and benign groups among the clinical characteristics (*p* = 0.036). No significant differences in lesion characteristics were observed between the groups in this subgroup (Table S1).

### 3.2. Comparison of Lesion-to-Normal Ratios (L/Ns) of Chromophores Between Malignant and Benign Lesions

**Figure 3:**
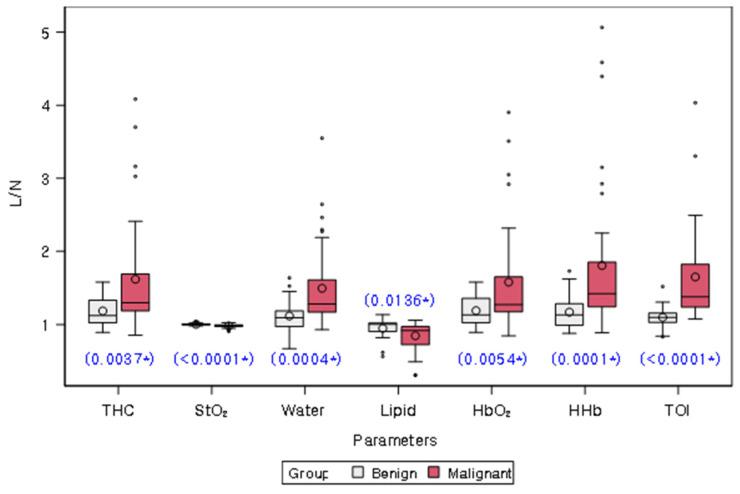
Distribution of lesion-to-normal ratios (L/Ns) of chromophores in malignancy and benign groups. White and red bars represent benign and malignant lesions, respectively. The mean value for each group is represented on the graph by a circle. The box in the plot represents the interquartile range, with the median value indicated by a line inside the box. The whiskers in the plot represent the range of values within a certain distance from the first and third quartiles, with any outliers indicated by dots or asterisks outside the whiskers. The blue star on the plot indicates a statistically significant difference between the two groups, as determined by a Wilcoxon rank sum test (*p* values provided). THC = total hemoglobin concentration, StO_2_ = percent oxygen saturation, Lipid = bulk lipid, HbO_2_ = oxy-hemoglobin, HHb = deoxy-hemoglobin, TOI = tissue optical index.

Table 2 and Figure 3 present the comparison of L/Ns of quantitative chromophores between malignant and benign groups. L/Ns of all chromophores differed significantly between the two groups (all *p* < 0.05). Specifically, L/Ns of THC, Water, HbO_2_, HHb, and TOI were significantly higher in the malignant group compared to the benign group, while L/Ns of StO_2_ and lipid were significantly lower in the malignant group.

Subgroup analysis of BI-RADS Category 4A cases revealed that L/Ns of all chromophores, except for Lipid, showed significant differences between malignant and benign lesions (all *p* < 0.05). Similar to the overall analysis, L/Ns of THC, Water, HbO_2_, HHb, and TOI were significantly higher in malignant lesions, while the L/N of StO_2_ was significantly lower in malignant lesions (Table S2, Figure S2).

### 3.3. Diagnostic Performance of Lesion-to-Normal Ratios (L/Ns) of Chromophores

**Figure 4:**
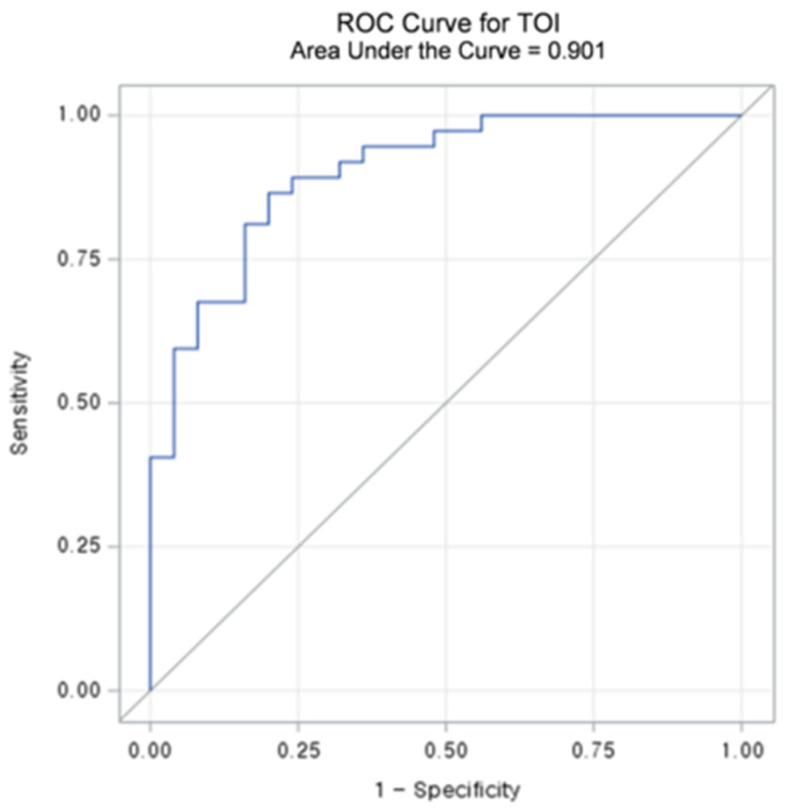
Receiver operating characteristic (ROC) curve and area under the curve (AUC) value of lesion to a normal ratio (L/N) of Tissue Optical Index (TOI).

**Figure 5:**
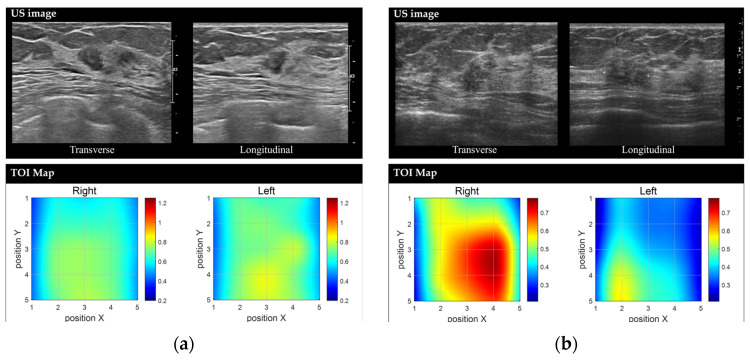
Representative images of benign and malignant breast lesions derived from the discrete multi-wavelength near-infrared spectrum (DMW-NIRS) imaging. (**a**) Conventional US image and Tissue Optical Index (TOI) map of a 9 mm mass in the left breast, located at the 2 o’clock position, 3 cm from the nipple, in a 50-year-old woman. The initial Breast Imaging Reporting and Data System (BI-RADS) category assessment by a breast-dedicated radiologist was 4A. The TOI_L/N_ ratio was 1.0544, below the threshold of 1.1862. A core needle biopsy revealed fibroadenomatoid hyperplasia. (**b**) Conventional US image and TOI map of a 13 mm mass in the right breast, located at the 10 o’clock position, 6 cm from the nipple, in a 53-year-old woman. The initial BI-RADS category assessment by a breast-dedicated radiologist was 4C. The TOI_L/N_ ratio was 1.5365, exceeding the threshold of 1.1862. A core needle biopsy confirmed invasive lobular carcinoma.

Table 3 summarizes the diagnostic performance metrics and threshold values for L/Ns of each chromophore. Among the chromophores, TOI_L/N_ exhibited the highest accuracy (83.9%) and sensitivity (86.5%). Regarding specificity, HHb_L/N_ demonstrated the best results (84.0%), followed by TOI_L/N_ (80.0%). The AUC-ROCs of L/Ns for the chromophores ranged from 0.686 to 0.901 (all *p* < 0.05). TOI_L/N_ achieved the highest AUC-ROC of 0.901 (95% CI: 0.825–0.976) with a threshold value of 1.1862 (Figure 4 and Figure 5), followed by StO_2_ L/N with an AUC-ROC of 0.796 (95% CI: 0.686–0.906).

Subgroup analysis of BI-RADS Category 4A cases is shown in Table S3. Among the chromophores, TOI_L/N_ exhibited the highest accuracy (90.6%), sensitivity (100.0%), and specificity (82.4%). AUC-ROCs of L/Ns of chromophores ranged from 0.612 to 0.902 (all *p* values except for lipid < 0.05). TOI_L/N_ achieved the highest AUC-ROC of 0.902 (95% CI: 0.788–1.000) with a threshold value of 1.1862, followed by HHb_L/N_ with an AUC-ROC of 0.824 (95% CI: 0.677–0.970).

### 3.4. Diagnostic Performance of Adaptive BI-RADS Categories Using Dmw-Nirs Results

Model 1, which adjusted BI-RADS categories based on TOI_L/N_ thresholds, achieved an AUC-ROC of 0.962 (95% CI: 0.923–1.000), significantly outperforming the prospectively assessed BI-RADS categories by breast-dedicated radiologists (AUC-ROC: 0.862, 95% CI: 0.796–0.929, *p* < 0.001). Model 2, which integrated radiologists’ reassessments of US findings with DOSI results, achieved an AUC-ROC of 0.922 (95% CI: 0.869–0.976), also significantly outperforming the retrospectively assessed BI-RADS categories by two breast-dedicated radiologists (K.M.J and J.Y) (AUC-ROC: 0.866, 95% CI: 0.807–0.925, *p* = 0.048).

For Model 2, when the two radiologists retrospectively assessed US images alone, each independently classified 29 of the 62 enrolled cases as BI-RADS Category 4A. After integrating the DMW-NIRS results, one radiologist downgraded five cases (17.2%) and the other downgraded four cases (13.8%) to BI-RADS Category 3. Among these downgraded cases, only one case per radiologist was ultimately diagnosed as malignant upon final pathology.

## 4. Discussion

Our study employed a novel DMW-NIRS technique to simultaneously and rapidly measure quantitative chromophores, demonstrating its predictive potential for malignant breast lesions. The L/N values of all chromophores derived from our method exhibited significant diagnostic utility. Notably, TOI_L/N_, previously proposed as an indicator of heightened metabolic activity [5,19], demonstrated the highest diagnostic accuracy (83.9%), sensitivity (86.5%), and AUC-ROC (0.901) among the DMW-NIRS parameters. Regarding specificity, TOI (80.0%) ranked second only to HHb (84.0%).

Several studies have utilized DOSI-derived functional information to distinguish malignant from benign breast lesions [20]. Consistent with these findings [13,14,15,21,22], our study confirmed that malignant lesions exhibit elevated water and hemoglobin levels, as well as decreased lipid content and tissue oxygen saturation compared to benign lesions. These differences likely reflect the increased cellularity, metabolism, and perfusion characteristics of malignant lesions. Interestingly, StO_2_ L/N demonstrated a notable AUC-ROC of 0.796 in our study, contrary to the findings by Leproux et al., where it had the lowest AUC-ROC (0.56) [15]. This discrepancy may result from differences in the study populations; our cohort included smaller malignant lesions (mean 22.29 mm, range 7–49 mm) compared to the larger palpable lesions (mean 26 mm, range 12–56 mm) examined in the previous study [15]. Further research is needed to investigate the relationship between hypoxia in malignant metabolism and StO_2_.

Compared to a prior study [15], our novel DMW-NIRS technique achieved similar diagnostic accuracy (69.4–83.9% in our study vs. 57.1–95.2% in the previous study) and AUC-ROC (0.69–0.90 in our study vs. 0.56–0.99 in the previous study). However, unlike the previous study that utilized lasers and broadband light in the near-infrared range of 650 nm to 1000 nm to a single spot on the breast tissue [15], our technique utilized LEDs with multiple wavelengths to simultaneously measure five spots on the breast tissue, significantly reducing measurement time (1/5 of the previous method, requiring just five minutes). This enhancement in efficiency and ease of use makes our approach highly suitable for clinical settings where timely and accurate diagnoses are essential.

Given the low sensitivity of mammography in women with dense breasts, supplemental US screening plays a significant role in detecting additional cancers [23,24]. However, its high false positive rate and the resulting benign biopsies remain challenges. In our study, 51.6% (32/62) of participants fell into Category 4A based on US findings. Within this subgroup, our DMW-NIRS system demonstrated excellent performance in differentiating malignant from benign lesions, with TOI_L/N_ achieving an AUC-ROC of 0.902 and a specificity of 82.4%. These findings suggest that DMW-NIRS could serve as a highly specific adjunct to address the limitations of breast US in low-suspicion BI-RADS categories. Previous studies, such as that by Zhu et al., have shown that DOSI can assist radiologists in reducing biopsy recommendations for BI-RADS 4A and 4B lesions [25]. Our findings support this potential, demonstrating the feasibility of rapid measurements with a DMW-NIRS system and its ability to enhance diagnostic accuracy while reducing unnecessary biopsies in BI-RADS 4A lesions.

We also developed two adaptive BI-RADS categories (Model 1 and Model 2) based on the DMW-NIRS results. Both models showed increased AUC-ROC (0.962 for Model 1 and 0.922 for Model 2) compared to the initial BI-RADS categories assessed by breast-dedicated radiologists (0.862 for prospective assessment and 0.866 for retrospective assessment). Notably, when the DMW-NIRS results were considered, one radiologist downgraded five cases (17.2%) and another downgraded four cases (13.8%) from BI-RADS Category 4A to Category 3. While these findings suggest that DMW-NIRS has the potential to reduce unnecessary biopsies by increasing PPVs for Category 4A lesions, it is essential to exercise caution. One lesion downgraded to Category 3 was ultimately diagnosed as malignant, underscoring the need for further research to identify specific scenarios where the DMW-NIRS may have limitations.

There are several limitations to our study. First, it was conducted at a single institution with a relatively small sample size (*n* = 62). However, compared to previous investigations, our study included a larger sample size, sufficient to demonstrate the feasibility of applying this new DMW-NIRS technique clinically. Second, measurements were performed by a single operator, introducing potential bias. Future studies should emphasize adequate training to standardize DMW-NIRS usage. Third, differences in patient and lesion characteristics between malignant and benign groups may have influenced the results, though these differences reflect real-world clinical scenarios and likely had minimal impact on the overall performance. Fourth, we excluded patients whose NAC was within the measurement range due to potential interference from pigmentation. Further studies should explore strategies to correct this limitation. Finally, the fixed source–detector separation of 30 mm in the DMW-NIRS system imposes a limitation on measurement depth, as near-infrared light typically penetrates to about half the source–detector separation (approximately 15 mm). Although most lesions in this study were within this depth range, the diagnostic reliability of the system may be compromised for lesions located deeper than 15 mm.

## 5. Conclusions

In conclusion, the L/N values of chromophores measured with our novel DMW-NIRS system show strong potential as an adjunct to BI-RADS categorization for distinguishing malignant from benign breast lesions. This technique provides a rapid, non-invasive, and practical method to enhance diagnostic accuracy, particularly for BI-RADS 4A lesions. While this study establishes the feasibility of the DMW-NIRS system, further validation with larger, more diverse patient cohorts is necessary to confirm its clinical utility and refine its application.

## Figures and Tables

**Table 1:** Comparison of clinical and lesion characteristics between malignancy and benign groups.

	Malignancy (*n* = 37)	Benign (*n* = 25)	*p* Value
Clinical characteristics			
Age (years, mean ± SD)	53.4 ± 10.4	44.9 ± 10.4	0.003
Range	33–80	29–71	
Age classification			0.011
<50 years	13 (35.1)	17 (68.0)	
≥50 years	24 (64.9)	8 (32.0)	
BMI (kg/m^2^, mean ± SD)	23.4 ± 3.0	23.5 ± 3.2	0.923
BMI classification			0.547
Underweight (<18.5 kg/m^2^)	1 (2.7)	1 (4.0)	
Healthy Weight (18.5 kg/m^2^~23 kg/m^2^)	19 (51.4)	9 (36.0)	
Overweight (23 kg/m^2^~25 kg/m^2^)	8 (21.6)	9 (36.0)	
Obesity (≥25 kg/m^2^)	9 (24.3)	6 (24.0)	
Family history of breast cancer			0.830
No	25 (69.4)	18 (72.0)	
Yes	11 (30.6)	7 (28.0)	
Unknown	1 (0.0)		
Breast density			1.000
A (entirely fatty) or B (scattered fibroglandular)	5 (13.5)	2 (10.5)	
C (heterogeneously dense) or D (extremely dense)	33 (86.5)	17 (89.4)	
Unavailable	0	6	
History of hormonal therapy			
No	33 (89.2)	25 (100.0)	0.141
Yes	4 (10.8)	0 (0.0)	
Menopausal status			0.006
Premenopausal	13 (35.1)	19 (76.0)	
Perimenopausal	7 (18.9)	1 (4.0)	
Postmenopausal	17 (46.0)	5 (20.0)	
Lesion characteristics			
Maximal tumor diameter (mm, mean ± SD)	22.3 ± 11.6	16.1 ± 8.4	0.044
Distance from the nipple			0.043
1~3 cm	14 (37.8)	16 (64.0)	
4~8 cm	23 (62.2)	9 (36.0)	
>9 cm	0 (0.0)	0 (0.0)	
Distance from the skin (mm, mean ± SD)	5.7 ± 2.8	7.5 ± 3.6	0.045
Breast thickness at the tumor site (mm, mean ± SD)	21.8 ± 6.8	19.3 ± 4.82	0.126
BI-RADS Category			<0.001
3	0 (0.0)	8 (32.0)	
4A	15 (40.5)	17 (68.0)	
4B	5 (13.5)	0 (0.0)	
4C	7 (18.9)	0 (0.0)	
5	10 (27.0)	0 (0.0)	

Note: Percentages are in parentheses. SD = standard deviation. BMI = body mass index. BI-RADS = Breast Imaging Reporting and Data System. *p* values comparing the difference between malignancy and benign groups were calculated using the chi-square test or Fisher’s exact test for categorical factors and Two-sample *t*-test or Wilcoxon rank sum test for continuous factors (except unavailable data).

**Table 2:** Comparison of lesion-to-normal ratio (L/N) of chromophores between malignancy and benign groups.

	Malignancy (*n* = 37)	Benign (*n* = 25)	*p* Value
THC_L/N_			
median (min, max)	1.30 (0.85, 4.08)	1.12 (0.89, 1.58)	0.004
StO_2-L/N_			
median (min, max)	0.99 (0.91, 1.03)	1.00 (0.98, 1.04)	<0.001
Water_L/N_			
median (min, max)	1.28 (0.93, 3.55)	1.09 (0.67, 1.64)	<0.001
Lipid_L/N_			
median (min, max)	0.92 (0.31, 1.06)	1.00 (0.56, 1.14)	0.012
HbO_2-L/N_			
median (min, max)	1.27 (0.85, 3.90)	1.13 (0.89, 1.58)	0.005
HHb_L/N_			
median (min, max)	1.42 (0.89, 5.06)	1.13 (0.88, 1.73)	<0.001
TOI_L/N_			
median (min, max)	1.38 (1.08, 4.03)	1.10 (0.83, 1.52)	<0.001

Note: Percentages are in parentheses. L/N = lesion-to-normal ratio. *p* values comparing the difference between malignancy and benign groups were calculated using Two-sample *t*-test or Wilcoxon rank sum test.

**Table 3:** Diagnostic performance of lesion-to-normal ratio (L/N) of chromophores.

Parameter	Threshold	Accuracy	Sensitivity	Specificity	AUC-ROC (95% CI)	*p* Value
THC_L/N_	1.1862	0.694	0.784	0.560	0.719 (0.591, 0.846)	<0.001
StO_2L/N_	0.9947	0.742	0.730	0.760	0.796 (0.686, 0.906)	<0.001
Water_L/N_	1.1531	0.790	0.838	0.720	0.769 (0.643, 0.894)	<0.001
Lipid_L/N_	0.9787	0.726	0.838	0.560	0.686 (0.546, 0.827)	0.016
HbO_2L/N_	1.1357	0.694	0.811	0.520	0.710 (0.582, 0.839)	0.002
HHb_L/N_	1.3541	0.726	0.649	0.840	0.788 (0.675, 0.901)	<0.001
TOI_L/N_	1.1862	0.839	0.865	0.800	0.901 (0.825, 0.976)	<0.001

Note: L/N = lesion-to-normal ratio. CI = confidence interval. AUC-ROC = area under the receiver operating characteristic curve. Accuracy, sensitivity, and specificity were calculated based on an optimal threshold that was generated by using the Youden index (J) method. *p* values of the likelihood ratio test are provided.

## Data Availability

The datasets used and/or analyzed during the current study are available from the corresponding author on reasonable request.

## Supplementary Materials

The following supporting information can be downloaded at: https://www.mdpi.com/article/10.3390/diagnostics15091067/s1, Table S1: Comparison of clinical and lesion characteristics between malignancy and benign groups (only BIRADS Category 4A cases), Table S2: Comparison of lesion-to-normal ratio (L/N) of chromophores between malignancy and benign groups (only BIRADS Category 4A cases), Table S3: Diagnostic performance of lesion to normal ratio (L/N) of chromophores (only BI-RADS category 4A cases). Figure S1: Predictive performance of machine learning models, Figure S2: Distribution of lesion-to-normal ratio (L/N) of chromophores in malignancy.

## Abbreviations

The following abbreviations are used in this manuscript:

BI-RADS	breast imaging reporting and data system
DOSI	diffuse optical spectroscopic imaging
NIR	near-infrared
NAC	nipple–areolar complex
ROI	region of interest
HbO_2_	oxy-hemoglobin
HHb	deoxy-hemoglobin
Water	water
Lipid	bulk lipid
THC	total hemoglobin concentration
StO_2_	percent oxygen saturation
TOI	tissue optical index
L/N	lesion-to-normal ratio
AUC-ROC	area under the receiver operating characteristic curve
