# Supplementary material for: Innovative Discrete Multi-Wavelength Near-Infrared Spectroscopic (DMW-NIRS) Imaging for Rapid Breast Lesion Differentiation: Feasibility Study
Source: Diagnostics (Basel). 2025 Apr 23;15(9):1067. doi: 10.3390/diagnostics15091067 (PMC12071914; doi:10.3390/diagnostics15091067)
Supplement: Supplementary file 1 [file diagnostics-15-01067-s001.zip › FigS1 caption_revised.pdf]

Figure S1. Predictive performance of machine learning models

The performance of machine learning models predicting TOI, water, THC, and lipid concentrations. Results are shown separately for training and test datasets, with R^2 values indicating high model accuracy. TOI = tissue optical index, THC = total hemoglobin concentration, Lipd = bulk lipid.
