# Supplementary material for: Innovative Discrete Multi-Wavelength Near-Infrared Spectroscopic (DMW-NIRS) Imaging for Rapid Breast Lesion Differentiation: Feasibility Study
Source: Diagnostics (Basel). 2025 Apr 23;15(9):1067. doi: 10.3390/diagnostics15091067 (PMC12071914; doi:10.3390/diagnostics15091067)
Supplement: Supplementary file 1 [file diagnostics-15-01067-s001.zip › FigS2 caption_revised.pdf]

Figure S2. Distribution of lesion to normal ratio (L/N) of chromophores in malignancy and benign groups (only BI-RADS category 4A cases)

White and red bars represent benign and malignant lesions, respectively. The mean value for each group is represented on the graph by a circle. The box in the plot represents the interquartile range, with the median value indicated by a line inside the box. The whiskers in the plot represent the range of values within a certain distance from the first and third quartiles, with any outliers indicated by dots or asterisks outside the whiskers. The blue star on the plot indicates a statistically significant difference between the two groups, as determined by a Wilcoxon Rank Sums test (*P* values provided). BI-RADS = Breast Imaging Reporting and Data System, THC = total hemoglobin concentration, StO2 = percent oxygen saturation, Lipid = bulk lipid, HbO2 = oxy-hemoglobin, HHb = deoxy-hemoglobin, TOI = tissue optical index.
