# Supplementary material for: Innovative Discrete Multi-Wavelength Near-Infrared Spectroscopic (DMW-NIRS) Imaging for Rapid Breast Lesion Differentiation: Feasibility Study
Source: Diagnostics (Basel). 2025 Apr 23;15(9):1067. doi: 10.3390/diagnostics15091067 (PMC12071914; doi:10.3390/diagnostics15091067)
Supplement: Supplementary file 1 [file diagnostics-15-01067-s001.zip › Table S1_revised.pdf]

Table S1. Comparison of clinical and lesion characteristics between malignancy and benign groups (only BIRADS category 4A cases)

	Malignancy (n=15)	Benign (n=14)	*P* Value
Clinical characteristics			
Age (years, mean±SD)	51.7±10.9	43.9±8.9	0.036
Range	33-68	29-63	
Age classification			0.169
<50 years	7 (46.7)	12 (70.6)	
≥50 years	8 (53.3)	5 (29.4)	
BMI (kg/m^2, mean±SD)	23.0±2.9	23.1±2.6	0.966
BMI classification			0.167
Underweight (<18.5 kg/m^2)	0 (0.0)	1 (5.9)	
Healthy Weight (18.5 kg/m2 ~ 23 kg/m^2)	9 (60.0)	5 (29.4)	
Overweight (23 kg/m2 ~ 25 kg/m^2)	2 (13.3)	7 (41.2)	
Obesity (≥25 kg/m^2)	4 (26.7)	4 (23.5)	
Family history of breast cancer			0.478
No	8 (57.1)	5 (29.4)	
Yes	6 (42.9)	12 (70.6)	
Unknown	1	0	
Breast density			1.000
A (entirely fatty) or B (scattered fibroglandular)	2 (13.3)	2 (15.4)	
C (heterogeneously dense) or D (extremely dense)	13 (86.7)	11 (84.6)	
Unavailable	0	4	
History of hormonal therapy			0.212
No	13 (86.7)	17 (100.0)	
Yes	2 (13.3)	0 (0.0)	
Menopausal status			0.657
Premenopausal	5 (33.3)	13 (76.5)	
Perimenopausal	4 (26.7)	1 (5.9)	
Postmenopausal	6 (40.0)	3 (17.7)	
Lesion characteristics			
Maximal tumor diameter (mm, mean±SD)	15.7±9.3	17.1±9.5	0.545
Distance from the nipple			0.288
1~3 cm	6 (40.0)	10 (58.8)	
4~8 cm	9 (60.0)	7 (41.2)	
Distance from the skin (mm, mean±SD)	6.3±3.2	8.5±3.8	0.094
Breast thickness at the tumor site (mm, mean±SD)	19.3±6.7	20.2±5.2	0.668

Note.—Percentages are in parentheses.

BI-RADS = Breast Imaging Reporting and Data System. SD = standard deviation. BMI = body mass index.

P values comparing the difference between malignancy and benign groups were calculated using Chi-square test or Fisher's exact test for categorical factors and Two sample t-test or Wilcoxon rank sum test for continuous factors (except unavaliable data).
