# Supplementary material for: Innovative Discrete Multi-Wavelength Near-Infrared Spectroscopic (DMW-NIRS) Imaging for Rapid Breast Lesion Differentiation: Feasibility Study
Source: Diagnostics (Basel). 2025 Apr 23;15(9):1067. doi: 10.3390/diagnostics15091067 (PMC12071914; doi:10.3390/diagnostics15091067)
Supplement: Supplementary file 1 [file diagnostics-15-01067-s001.zip › Table S2_revised.pdf]

Table S2. Comparison of lesion to normal ratio (L/N) of chromophores between malignancy and benign groups (only BIRADS category 4A cases)

	Malignancy (n=15)	Benign (n=17)	*P* Value
$THC_{L/N}$			
median (min, max)	1.40 (1.05, 4.08)	1.12 (0.89, 1.58)	0.010
$StO_{2-L/N}$			
median (min, max)	1.00 (0.96, 1.03)	1.00 (0.98, 1.04)	0.032
$Water_{L/N}$			
median (min, max)	1.24 (0.93, 2.30)	1.12 (0.67, 1.64)	0.026
$Lipid_{L/N}$			
median (min, max)	0.95 (0.65, 1.06)	1.00 (0.56, 1.14)	0.290
$HbO_{2-L/N}$			
median (min, max)	1.40 (1.03, 3.90)	1.12 (0.89, 1.58)	0.014
$HHb_{L/N}$			
median (min, max)	1.40 (1.10, 5.06)	1.13 (0.88, 1.73)	0.002
$TOI_{L/N}$			
median (min, max)	1.28 (1.19, 4.03)	1.08 (0.83, 1.52)	<0.001

Note.—Percentages are in parentheses.

BI-RADS = Breast Imaging Reporting and Data System. L/N = lesion to normal ratio.

P values comparing the difference between malignancy and benign groups were calculated using Two sample t-test or Wilcoxon rank sum test.
