# Supplementary material for: Innovative Discrete Multi-Wavelength Near-Infrared Spectroscopic (DMW-NIRS) Imaging for Rapid Breast Lesion Differentiation: Feasibility Study
Source: Diagnostics (Basel). 2025 Apr 23;15(9):1067. doi: 10.3390/diagnostics15091067 (PMC12071914; doi:10.3390/diagnostics15091067)
Supplement: Supplementary file 1 [file diagnostics-15-01067-s001.zip › Table S3.pdf]

Table S3. Diagnostic performance of lesion to normal ratio (L/N) of chromophores (only BI-RADS category 4A cases)

Parameter	Threshold	Accuracy	Sensitivity	Specificity	AUC-ROC (95% CI)	*P* Value
$THC_{L/N}$	1.1862	0.750	0.933	0.588	0.769 (0.603, 0.934)	0.005
$StO_{2\text{-}L/N}$	0.9947	0.656	0.467	0.824	0.710 (0.520, 0.899)	0.024
$Water_{L/N}$	1.1531	0.750	0.800	0.706	0.733 (0.553, 0.914)	0.041
$Lipid_{L/N}$	0.9787	0.656	0.800	0.529	0.612 (0.406, 0.818)	0.648
$HbO_{2\text{-}L/N}$	1.1357	0.719	0.933	0.529	0.757 (0.589, 0.925)	0.008
$HHb_{L/N}$	1.3541	0.781	0.733	0.824	0.824 (0.677, 0.970)	<0.001
$TOI_{L/N}$	1.1862	0.906	1.000	0.824	0.902 (0.788, 1.000)	<0.001

Note.— BI-RADS = Breast Imaging Reporting and Data System. L/N = lesion to normal ratio. AUC-ROC = area under the receiver operating characteristic curve. CI = confidence interval. Accuracy, sensitivity, and specificity were calculated based on an optimal threshold that was generated by using the Youden index (J) method.

P values of the likelihood ratio test are provided.
