# Supplementary figures and images for: Innovative Discrete Multi-Wavelength Near-Infrared Spectroscopic (DMW-NIRS) Imaging for Rapid Breast Lesion Differentiation: Feasibility Study
Source: Diagnostics (Basel). 2025 Apr 23;15(9):1067. doi: 10.3390/diagnostics15091067 (PMC12071914; doi:10.3390/diagnostics15091067)

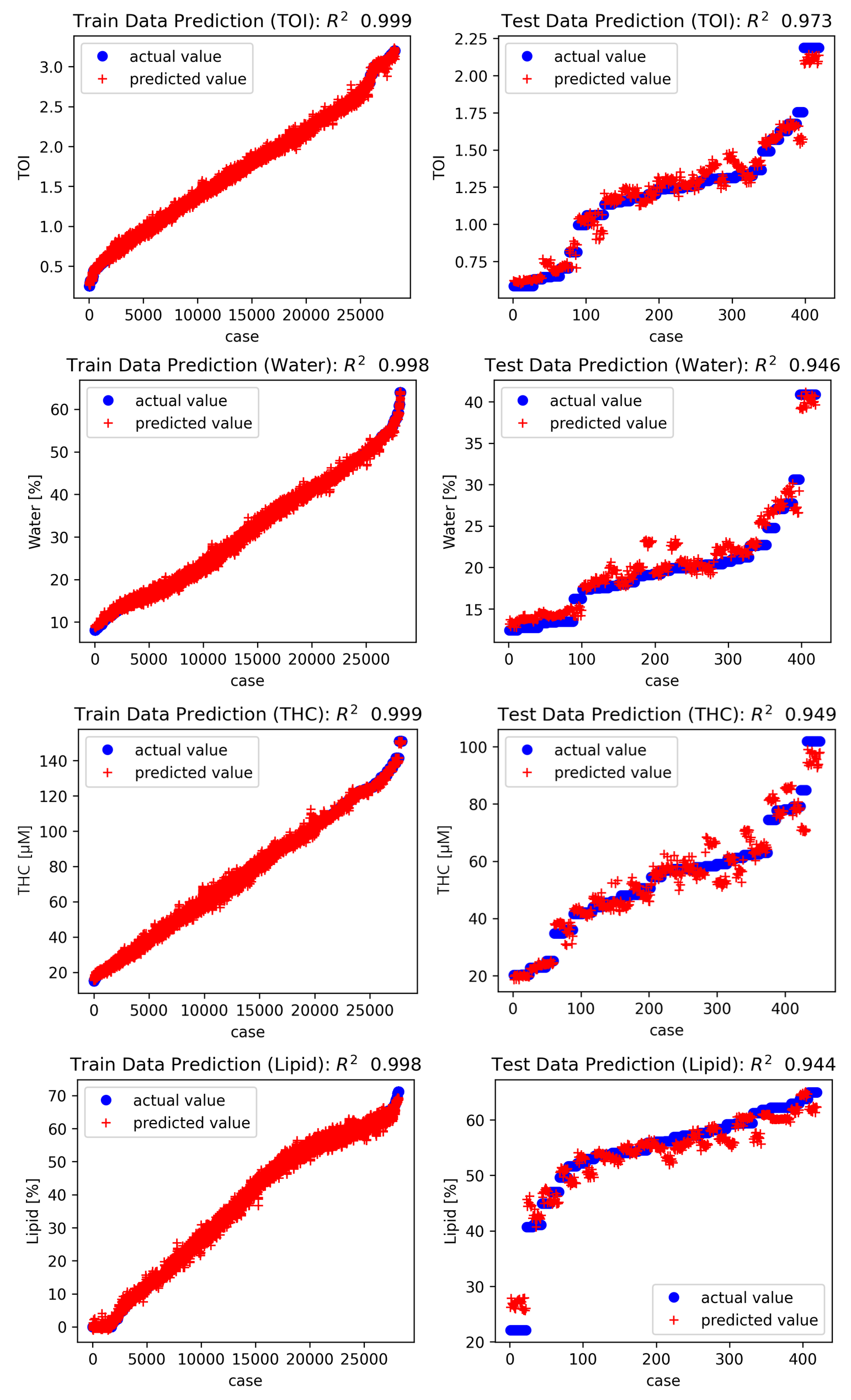

Supplement: Supplementary file 1 [file diagnostics-15-01067-s001.zip › FigS1.tif]

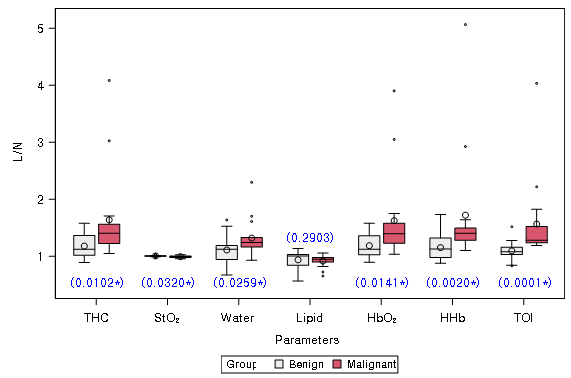

Supplement: Supplementary file 1 [file diagnostics-15-01067-s001.zip › FigS2.tif]
